# Current Status of the Sm14/GLA-SE Schistosomiasis Vaccine: Overcoming Barriers and Paradigms towards the First Anti-Parasitic Human(itarian) Vaccine

**DOI:** 10.3390/tropicalmed3040121

**Published:** 2018-11-21

**Authors:** Miriam Tendler, Marília S. Almeida, Monica M. Vilar, Patrícia M. Pinto, Gabriel Limaverde-Sousa

**Affiliations:** FIOCRUZ—Instituto Oswaldo Cruz, Laboratório de Esquistossomose Experimental, Av. Brasil, 4365, Manguinhos, Rio de Janeiro 21045-900, Brazil; sirianni@ioc.fiocruz.br (M.S.A.); mvilar@ioc.fiocruz.br (M.M.V.); pmpinto@ioc.fiocruz.br (P.M.P.); gabriel.sousa@ioc.fiocruz.br (G.L.-S.)

**Keywords:** schistosomiasis, vaccine, Sm14, FABP

## Abstract

Schistosomiasis, a disease historically associated with poverty, lack of sanitation and social inequality, is a chronic, debilitating parasitic infection, affecting hundreds of millions of people in endemic countries. Although chemotherapy is capable of reducing morbidity in humans, rapid re-infection demonstrates that the impact of drug treatment on transmission control or disease elimination is marginal. In addition, despite more than two decades of well-executed control activities based on large-scale chemotherapy, the disease is expanding in many areas including Brazil. The development of the Sm14/GLA-SE schistosomiasis vaccine is an emblematic, open knowledge innovation that has successfully completed phase I and phase IIa clinical trials, with Phase II/III trials underway in the African continent, to be followed by further trials in Brazil. The discovery and experimental phases of the development of this vaccine gathered a robust collection of data that strongly supports the ongoing clinical phase. This paper reviews the development of the Sm14 vaccine, formulated with glucopyranosyl lipid A (GLA-SE), from the initial experimental developments to clinical trials including the current status of phase II studies.

## 1. Introduction

Schistosomiasis is the second-most socioeconomically devastating parasitic disease after malaria. The disease is both chronic and debilitating with an estimated 200 million people infected, most of whom (85%) live in Africa. Of those infected, 120 million are symptomatic and 20 million present severe disease symptoms [1]. These estimates may err on the low side since meta-analysis has found the number of people at risk to be closer to 800 million [2]. Globally, the impact of schistosomiasis remains high and the estimated number of disability-adjusted life years (DALYs) has increased with the inclusion of previously under-recognized morbidities not previously included in the DALY index (for example, stunted growth and anemia associated with retarded intellectual development) in infants, toddlers and school age children, whose physical health and intellectual capacity are fundamental to nation development and sustainability [3,4]. In addition, high-definition circulating antigen tests, such as the POC-CCA assay, indicate that the current number of infections may be at least 10 times higher than that shown by egg detection [5]. In Brazil, the largest endemic country for schistosomiasis, 6 million individuals are estimated to be infected and 25 million are at risk of contracting the disease [6,7].

Mass chemotherapy has been the strategy of choice for the control schistosomiasis with the support of international health funding agencies. Estimates show that at least 206.5 million people were treated in 2016 [8]. However, the strategy of large-scale chemotherapeutic treatment, also equivocally called ‘prophylactic treatment’, over a period of 30 years has failed to control schistosome transmission. Approximately 300 million US dollars are being spent annually on treatments applied to the same populations year after year with no prospect of preventing reinfection or the need for repeat treatments [9]. ‘Deworming’ initiatives, originally applied to animal species only, were proposed as a tool for schistosomiasis control programs focused on school children in endemic countries [10,11].

In the veterinary field, under One Health policies for the control of helminth infections such as *Fasciola hepatica*, the major parasitic infection of livestock worldwide, there is a strong demand for the replacement of anti-helminthic drugs with vaccines. This would significantly reduce the amount of chemical residues in meat, milk and added-value products. Indeed, vaccines are considered to be the most environmentally- and human health-friendly method of control of fascioliasis in livestock [12].

The potential introduction of vaccines into schistosomiasis control programs brings hope to the poor living in endemic areas. The Brazilian Sm14 vaccine project was launched in the 1990s and strongly pushed in the context of a formal WHO program aimed at the development of an anti-schistosomiasis vaccine. The main outcome of this initiative was the selection of six priority antigen candidates of which only Sm14, continues to be developed (Table 1, adapted from [13]).

With strategic support from WHO, the Sm14 vaccine, which is based on a recombinant protein, is moving forward in an endemic country towards final development. It is being developed utilizing sophisticated and modern technological platforms and professionally conducted within a network of outstanding companies and collaborators. It is the result of long-term scientific developments carried under the coordination of FIOCRUZ, a public institution linked to the Brazilian Ministry of Health and is protected by strong patents, owned by FIOCRUZ, in all countries of interest worldwide.

Recently, the Consultative Expert Working Group on Research and Development: Financing and Coordination (CEWG/WHO) selected the Sm14 vaccine as one of six demonstration projects, globally, following an extensive review process. It was recognized as being fully in accordance with the principles of CEWG, such as clear mechanisms of de-linkage of costs from investments in research and development (R&D) from costs of final product, accessibility, affordability, viability and open knowledge innovation. The CEWG recognized that the Sm14 vaccine may become a key tool for the implementation of effective infection reduction and transmission control programs for schistosomiasis that would not rely only on chemotherapy [14]. 

**Figure 1:**
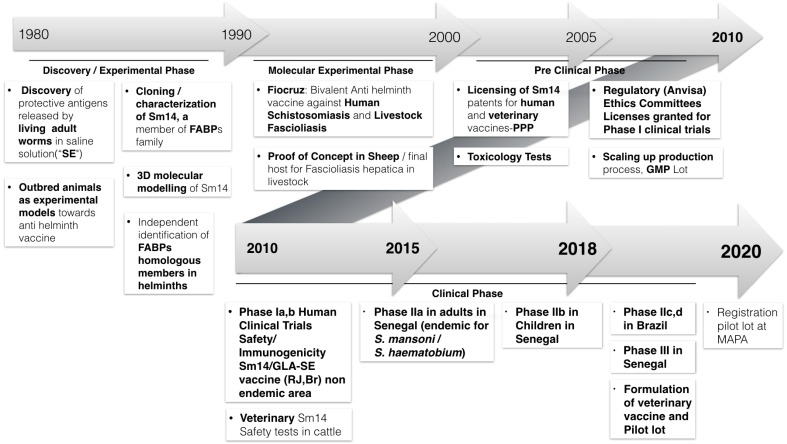
Timeline: Sm14/GLA-SE anti-schistosomiasis vaccine - from discovery phase to final product. MAPA: Brazilian Ministry of Agriculture.

Over recent years it has been possible to complete important milestones in the development of the vaccine including scaling up production process from laboratory bench to a clinical trial scale as well as the successful conclusion of two phase I human trials in healthy adults (male and female) living in a Brazilian non-endemic area (2011–2014) [15] together with a first phase II trial in 30 male adults living in highly endemic area for both *Schistosoma mansoni* and *S. haematobium* at the Senegal River Basin (2015–2017). In the latter, safety was extensively confirmed and strong and long-lasting immunogenicity was also demonstrated (manuscript in preparation). Master cell bank generation has recently been completed and production in good manufacturing practices (GMP) of a second Sm14 lot is currently ongoing, under the coordination of the Infectious Disease Research Institute (IDRI, Seattle, US). This article is an overview of the development of the Sm14 schistosomiasis vaccine development from antigen discovery to the current human studies (Figure 1). The need to overcome barriers for the establishment of a truly humanitarian vaccine—addressing the needs of the developing world—will be discussed.

## 2. Innovative Strategies Adopted for Antigen Discovery and Early Development Phases

Biotechnological advances in various areas of vaccine research have contributed to the development of safer and more effective formulations. Efforts to develop anti-helminth vaccines have been ongoing for many years and are continuing to progress in the identification of candidate antigens. These efforts have recently been boosted by the generation of a number of helminth genome sequences [16]. The development of a vaccine against schistosomiasis would represent an important step in the context of research and development in public health for poor populations infected and exposed to schistosomes. There have been initiatives to develop a vaccine against schistosomiasis from research groups in different countries. The Brazilian Sm14-based anti-schistosomiasis vaccine is the only one of these initiatives that is currently undergoing clinical trials [17].

In contrast to the current ‘OMICS’ strategies, in which high-throughput screening of potential target antigens are processed in parallel by automated ‘discovery protocols and platforms’, the Sm14 project was initiated by gathering observations from animal models of infection together with logical progression based on experimental evidence. 

The first original approach was in the methodology for generating a worm extract that was subsequently used to assess protective immunity. Rather than lyophilized parasites, generally used by other research groups, a saline extract containing secreted/excreted antigens derived from living adult schistosomes was utilized for initial immunizations. The restricted number of potential protective antigens released in this saline extract allowed an accelerated identification of strong antigen candidates for molecular analysis and gene cloning [18,19,20,21,22]. Innovative methods were also adopted for the assessment of immunity, where the use of outbred models, in contrast to the commonly-employed inbred animals, allowed a better representation of the ultimate target population and provided a unique opportunity to develop an alternative strategy for the assessment of protective immunity. The analyses were stratified based on the measurement of frequencies of worm burdens within vaccinated animal populations as compared with non-vaccinated infected controls, as opposed to evaluation of mean values of parasite loads, as usually adopted. A solid base of pre-clinical data was generated establishing the immunization protocols that would be adopted during the following steps of the development [23,24,25,26,27,28,29,30,31]. 

As molecular biology tools evolved and gene-cloning techniques became available, several antigens released in the saline culture medium of live schistosomes, were cloned and sequenced. Serum from rabbits immunized with the ‘saline extract’ was used to screen an adult *S. mansoni* cDNA library and the most promising antigen was identified as a member of the fatty acid binding protein (FABP) family, termed Sm14 [32]. Molecular modeling studies from our group predicted the beta-barrel structure of the Sm14 [33] that was later experimentally confirmed by crystallography [34] and nuclear magnetic resonance [35]. These analyses allowed the engineering of a stabilizing mutation that conferred a remarkable long-term stability, while maintaining the function and immunogenicity of the antigen [36].

Sm14 was shown to be particularly important to helminths, which are not capable of synthetizing fatty acids but rely on these being provided by the host species. Lipids, apart from being constituents of membranes, have important roles in development of different lifecycle stages and the evasion of immune responses both by adult worms and larvae [37]. In addition to the publication describing the a FABP family member in *Schistosoma mansoni* [32], different groups published the identification of homologous FABP protein members from FABP family in many helminths of human and veterinary importance. Of particular importance was the identification of the *F. hepatica* FABP [38]. *F. hepatica* is the main parasite of livestock worldwide. We have managed to successfully test Sm14 vaccination against *F. hepatica* in mice, followed by two independent experiments in sheep, one of the definitive host species for fascioliasis [39]. These experiments that demonstrated that Sm14 is also protective against *F. hepatica* infection has led to Sm14 being developed in parallel as the molecular basis for a veterinary vaccine by FIOCRUZ, in collaboration with the private Brazilian company Ourofino Animal Health. 

## 3. Clinical Studies

The licensing of the Sm14 patents for veterinary use gave birth to a public–private partnership (PPP) model of product development that rendered significant visibility to the Sm14 vaccine project. Such a gain in momentum was followed by strong support for human vaccine development by the Brazilian government project financing agency (FINEP) that allowed the use of contract research organizations (CROs) for antigen production, quality control and fill-finish in world-class academic GMP facilities based in the United States of America in collaboration with the Ludwig Institute for Cancer Research, Cornell University and the Infectious Disease Research Institute (IDRI, Seattle, USA).

In order to have a consistent, stable and defined final product for clinical human use, the Sm14 antigen was formulated with the synthetic adjuvant glucopyranosyl lipid A (GLA-SE), a clinically-approved molecule already used in a number of commercially-available human vaccines.

In December of 2010, the Brazilian Health Regulatory Agency (ANVISA) approved a phase Ia clinical trial in 20 healthy male volunteers from a non-endemic area (Rio de Janeiro, Brazil) to evaluate the safety of the investigational product. The study was conducted by the Brazilian National Institute of Infectious Diseases (INI/FIOCRUZ). The results of this first trial demonstrated the safety of the vaccine in the studied population, showing no systemic reactogenicity. No serious adverse events were associated to the investigational product [15]. A phase Ib clinical study, to evaluate the safety and immunogenicity of the vaccine preparation in 10 healthy women volunteers, was also then successfully concluded in 2012 (manuscript in preparation).

In 2015–2016, within the scope of a CEWG Demonstration Project, a phase IIa trial was developed in 30 male adults living in a highly endemic area for both *S. mansoni* and *S. haematobium* in the Senegal River Basin. The trial was conducted by a specialized team from Espoir Pour La Santé (EPLS), linked to the Pasteur Institute of Lille (IPL, France), headed by Dr. Gilles Riveau (IPL) in conjunction with the FIOCRUZ group and the Brazilian biotechnology company Orygen Biotecnologia the license holder of Sm14 for human use (ClinicalTrials.gov Identifier: NCT03041766) [40]. The main objectives of the trial were safety and immunogenicity of the Sm14/GLA-SE vaccine. These objectives were fully achieved. The investigational product rSm14 (50 µg) formulated with GLA-SE in two dosages (2.5 µg and 5 µg/dose, denominated groups 1 and 2, respectively) and administered intramuscular (IM), was shown to be safe with no observed serious adverse events in either group. The most common reactions were local pain and heaviness of the vaccinated arm. These reactions were transitory and mild. Seroconversion of 92% of individuals after the second dose were observed, similar to the pattern observed in the phase I trials [15]. Immunogenicity based on additional cellular responses, memory cells and T cell activation markers were analyzed at IDRI in an extensive panel focusing on the identification of a vaccine-related immune response.

After the completion of the phase IIa trial and based on the observed induction of a strong and long-lasting immune response, an extension study to assess the possible persistence and profile of this response beyond the initial schedule of the trial was duly authorized by Senegalese Ministry of Health (MoH). This was carried out between August and December 2017 with the inclusion of two additional time points, 9 and 12 months, after the first vaccine injection. ELISA tests performed at Centre de Recherche Biomédicale/Espoir Pour La Santé (EPLS) showed the persistence of significant specific antibody titers up to 12 months after the first vaccination dose with Sm14/GLA-SE (manuscript in preparation).

A phase IIb trial design and protocol were defined in January 2018, based on the results of the phase IIa trial in adults. The phase IIb trial will involve 95 school children from 7–11 years of age living in the same endemic area for both *S. mansoni* and *S. hamatobium* of the Senegal River Basin region. Ethical committee approval and regulatory approval from the Senegalese MoH have been obtained. EPLS has already initiated the trial in the same region as the original phase IIa trial in adults, using the same lot of GMP Sm14 under a regimen of three IM doses of 50 µg/dose formulated with 2.5 µg of GLA-SE, 30 days apart. The phase IIa and b trials are largely funded by Orygen Biotecnologia in conjunction with FIOCRUZ and with support of CEWG-WHO platform and financial fund.

## 4. Sm14 +GLA-SE: A Humanitarian Anti-Schistosomiasis Vaccine

During the process of analysis by WHO member state representatives, within a regional structure, and extensive subsequent analysis by technical experts and ad hoc committees, the Sm14 schistosomiasis vaccine, was selected by the WHO Executive Board as one of the current list of six demonstration projects. A stakeholders meeting organized by WHO at its Geneva headquarters was held in June 2015 prior to the release of the first installment of funding. During the process of selection and shortlisting of the candidate demonstration projects presented projects, discussions on the scientific merits, state of the art of the project and requirement for full adherence to CEWG principles, much was learned concerning the mandatory need to assure the accessibility and affordability of this vaccine to the poor endemic countries in which it will ultimately be used [14]. 

From its inception, the Sm14 schistosomiasis vaccine has been designed to be both effective and low-cost. To achieve this goal, several innovations for vaccine development were implemented and a strong effort was made to choose intellectual property-free components [36]. This has led to the successful development of a very low-cost, stable product involving a large-scale production process.

De-linkage of final product price from the costs of the long R&D phases has been achieved by Sm14 being initially developed at a governmental scientific foundation (FIOCRUZ) with support from funds from public Brazilian financial institutions (FINEP and FAPERJ).

After 2005, licensing of FIOCRUZ patent rights for human use to a private Brazilian company was through contracts designed to protect the accessibility, affordability and supply strategies for the lower- and middle-income countries (LMIC) that are the target areas to receive the anti-schistosomiasis vaccine. The presently licensee company, Orygen Biotecnologia, is a startup company wholly owned by two of the largest Brazilian pharmaceutical companies. Its involvement in the final development of the Sm14 human vaccine is crucial. The company has agreed to a cost plus pricing strategy, as adopted by WHO for vaccine pricing [41]. 

In parallel, the veterinary anti-fasciola vaccine is being developed in keeping with current European guidelines to reduce the presence of anti-helminthic drug chemical residues in milk and meat of livestock. It is aimed at rich countries and markets and designed to contribute/support future potential large-scale delivery programs. 

We are no longer at a stage when an anti-schistosomiasis vaccine is to be discussed, attacked or delayed, as it was for decades along with all anti-parasite vaccines. Our knowledge concerning vaccines has improved enormously, as have the technical resources available. Vaccines represent the intervention strategy with the best cost–benefit ratio thus far applied in public health. Moreover, transmission control of infectious/transmissible diseases has only been achieved through vaccination. Sanitation, chemotherapy and health education are not sufficient to eliminate parasitic diseases that disproportionally affect people living in poor countries. Immunization with a safe and effective vaccine can contribute to a long-term reduction of schistosome egg excretion from the host, thus truly controlling transmission. So far, there are no vaccines against the parasites that afflict countries fighting to emerge from poverty and reach better conditions of health and overall development. 

The Sm14 vaccine against schistosomiasis is being developed as a humanitarian vaccine to be included in effective schistosomiasis transmission control programs that will hopefully invert the paradigm for a north-to-south route for technology generation and contribute to the broad use of the most safe, effective and environmentally and human-friendly health-promoting strategy, prophylactic vaccination.

## Figures and Tables

**Table 1:** Schistosomiasis priority antigens selected by the WHO for independent testing (adapted from [13]).

Antigen	Size (kDa)	Stage Expressed	Description	Protection (%)	Place of Development
Glutathione S-transferase (P28/GST)	28	Adult/somula/egg	Enzyme	30–60	Institut Pasteur, Lille, France
Paramyosin (Sm97)	97	Adult/somula	Muscle protein	30	Case Western Reserve University/National Institute of Health/Cornell University, USA
IrV-5	62	Adult/somula/egg	Muscle protein	50–70	Johns Hopkins School of Medicine, Baltimore, USA
Triose phosphate isomerase (TPI)	28	Adult/somula/egg	Enzyme	30–60	Harvard School of Public Health, Boston, USA
Sm23	23	Adult/somula/egg	Integrated membrane protein	40–50	Johns Hopkins School of Medicine/Harvard School of Public Health, USA
Sm14	14	Adult/somula	Fatty acid-binding protein	65	Instituto Oswaldo Cruz, Rio de Janeiro, Brazil
