# Correction: Tendler, M., et al. Current Status of the Sm14/GLA-SE Schistosomiasis Vaccine: Overcoming Barriers and Paradigms towards the First Anti-Parasitic Human(itarian) Vaccine. *Trop. Med. Infect. Dis.* 2018, *3*, 121

**DOI:** 10.3390/tropicalmed4010016

**Published:** 2019-01-19

**Authors:** Miriam Tendler, Marília S. Almeida, Monica M. Vilar, Patrícia M. Pinto, Gabriel Limaverde-Sousa

**Affiliations:** FIOCRUZ—Instituto Oswaldo Cruz, Laboratório de Esquistossomose Experimental, Av. Brasil, 4365, Manguinhos, Rio de Janeiro 21045-900, Brazil; sirianni@ioc.fiocruz.br (M.S.A.); mvilar@ioc.fiocruz.br (M.M.V.); pmpinto@ioc.fiocruz.br (P.M.P.); gabriel.sousa@ioc.fiocruz.br (G.L.-S.)

The authors wish to make the following correction to this paper [1]:

Where it reads:

“In order to have a consistent, stable and defined final product for clinical human use, the Sm14 antigen was formulated with the synthetic adjuvant glucopyranosyl lipid A (GLA-SE), a clinically-approved molecule already used in a number of commercially-available human vaccines.”

It should read:

“In order to have a consistent, stable and defined final product for clinical human use, the Sm14 antigen was formulated with the synthetic adjuvant glucopyranosyl lipid A (GLA-SE), an adjuvant successfully tested in clinical trials with different human vaccine candidates.”

The authors would like to apologize for any inconvenience caused to the readers by this change.
